# Optimization of an *E. coli *L-rhamnose-inducible expression vector: test of various genetic module combinations

**DOI:** 10.1186/1472-6750-8-2

**Published:** 2008-01-14

**Authors:** Angelika Wegerer, Tianqi Sun, Josef Altenbuchner

**Affiliations:** 1 Institut für Industrielle Genetik, Universität Stuttgart, Allmandring 31, 70569 Stuttgart, Germany

## Abstract

**Background:**

A capable expression vector is mainly characterized by its production efficiency, stability and induction response. These features can be influenced by a variation of modifications and versatile genetic modules.

**Results:**

We examined miscellaneous variations of a *rhaP*_*BAD *_expression vector. The introduction of a stem loop into the translation initiation region of the *rhaP*_*BAD *_promoter resulted in the most significant improvement of *eGFP *expression. Starting from this plasmid, we constructed a set of expression vectors bearing different genetic modules like *rop*, *ccdAB*, *cer *and combinations thereof, and tested the efficiency of expression and plasmid stability. The plasmid pWA21, containing the stem loop, one *cer *site and *rop*, attained high expression levels accompanied by a good stability, and on that score seems to be a well-balanced choice.

**Conclusion:**

We report the generation of variations of the *rhaP*_*BAD *_expression vector and characterization hereof. The genetic modules showed a complex interplay, therefore two positive effects combined sometimes resulted in a disadvantage.

## Background

The demands on a valuable expression system are, in a nutshell, to receive high product yields, to provide a stable and tightly regulatable expression and to ensure high plasmid stability. The properties of such a system are determined by the combination of a specific organism with the desired gene, whereas the plasmid features play a crucial role, too. The effects of two or more determinants can not easily be estimated by addition of singular effects, because combined features can cancel each other out. Therefore, several expression systems have to be tested and the conditions have to be adjusted.

Owing to *Escherichia coli *being a model organism for genetic studies, a multitude of well-established regulatable promoters are available. A distinction is drawn between positively and negatively controlled regulatory mechanisms. For many promoters, especially those involved in carbohydrate catabolism, both possibilities are implemented, which is true for the well characterized *lac-*operon for instance. In other cases, such as the L-arabinose operon or the L-rhamnose operon, the expression is positively regulated. These systems are often characterized by a slower response with very low basal transcriptional activity, which can be a great advantage for the production of proteins that are detrimental to the host cell. The L-rhamnose system has successfully been used to express a variety of genes [1-3]. This system often provides better results compared to other vectors, especially if the expression of a gene usually leads to a large moiety of insoluble protein.

L-rhamnose is taken up by the RhaT transport system, converted to L-rhamnulose by an isomerase (RhaA) and then phosphorylated by a kinase (RhaB). Subsequently, the resulting rhamnulose-1-phosphate is hydrolyzed by an aldolase (RhaD) into dihydroxyacetone phosphate, which is metabolized in glycolysis, and L-lactaldehyde. The latter can be oxidized into lactate under aerobic conditions and be reduced into L-1,2-propanediol under unaerobic conditions. The genes *rhaBAD *are organized in one operon which is controlled by the *rhaP*_*BAD *_promoter. This promoter is regulated by two activators, RhaS and RhaR, and the corresponding genes belong to one transcription unit which is located in opposite direction of *rhaBAD*. If L-rhamnose is available, RhaR binds to the *rhaP*_*RS *_promoter and activates the production of RhaR and RhaS. RhaS together with L-rhamnose in turn binds to the *rhaP*_*BAD *_and the *rhaP*_*T *_promoter and activates the transcription of the structural genes. However, for the application of the rhamnose expression system it is not necessary to express the regulatory proteins in larger quantities, because the amounts expressed from the chromosome are sufficient to activate transcription even on multi-copy plasmids. Therefore, only the *rhaP*_*BAD *_promoter has to be cloned upstream of the gene that is to be expressed. Full induction of *rhaBAD *transcription also requires binding of the CRP-cAMP complex [4], which is a key regulator of catabolite repression.

In addition to transcriptional regulation, the degradation of messenger RNA (mRNA) as well as translation initiation appear to be important factors in controlling the level of gene expression. Most bacterial mRNAs show a high turnover rate which allow to rapidly adjust gene expression to the specific needs of the cells. RNase E is the principal endonuclease involved in mRNA decay in *E. coli*. The action of RNase E is favored by an accessible 5' terminus carrying a monophosphate residue [5]. Therefore, sequence independent thermodynamically stable 5'stem-loop structures protect mRNA from endonucleolytic attack by RNase E as seen in *ompA *or T7 gene *10 *mRNA which show unusual long half-lifes [6,7]. Translation initiation is greatly enhanced in *E. coli *and other bacteria by the Shine-Dalgarno sequence (SD) in mRNA, located 5–9 base pairs upstream of the initiation codon [8]. The canonical sequence (5'-AAGGAGG-3') is complementary to a sequence close to the 3' end of the 16 S rRNA. Numerous studies suggest, that mRNA translation is less efficient when the SD sequence has a lower degree of complementarity to the 16 S rRNA or a different distance to the start codon [9].

Since a read-through by the RNA polymerase can lead to severe instability of the expression system, it is recommended to insert a transcription terminator downstream of the desired gene. The vectors used in this study contain a Rho-independent terminator from the *rrnB *operon, which forms a stem-loop structure. Besides the transcription termination, the stability of a vector can be affected by miscellanous parameters.

The multimerization of plasmids reduces the copy number per cell and leads to segregational instability, a phenomenon known as dimer catastrophe [10]. Multimers can be resolved to monomers by site-specific recombination via the Xer-*cer *System of ColE1 [11]. Additionally, the promoter P_*cer *_within *cer *directs the synthesis of the 90 nt transcript Rcd (regulator of cell division), whose overexpression strongly inhibits the growth of cells on solid media, whereas in broth culture the growth is slowed down but not stopped [12,13]. Thus Rcd might be part of a checkpoint which causes a delay in cell division until multimers are converted into monomers, and Rcd seems to play a role in plasmid maintenance, which is functionally independent of dimer resolution [14]. Dimer-containing cells grow more slowly than their monomer-containing counterparts, and the appearance of Rcd correlates with the inhibition of division of multimer-containing cells, perhaps in order to provide the opportunity to resolve the multimers [15]. Due to the fact that an antisense target on the *E. coli *chromosome could not be found, it has been suggested, that Rcd might interact directly with a protein target [16].

Another parameter that can be of importance to the expression output is the number of plasmids contained by the cells. In ColE1-type plasmids an efficient regulation mechanism has been evolved, that helps to maintain a constant copy number by counteracting occasional deviations from the steady state level. This inhibitor-target mechanism is based on the negative control of the frequency of replication initiation events, mediated by the interaction of two RNA molecules, RNAI and RNAII and Rop (repressor of primer), a protein consisting of 63 amino acids. In this system RNAII is a preprimer whose processing into a primer by RNase H is inhibited by the hybridization with RNAI. Additionally, the interaction of target and inhibitor is enforced by Rop, and in absence of Rop the copy number is quintupled [17,18].

Beside the possibility to ensure plasmid maintenance by an improved stability, this task can also be performed by killing or inhibiting all cells which have lost their plasmids. Beyond the application of antibiotics combined with resistance markers on the plasmids, this function can be fulfilled by so called addiction modules, which induce programmed cell death in case of loss. This genetic system consists of two components, a stable toxin and an unstable antitoxin. Such systems were mainly found in low-copy plasmids of *E. coli*, where cured cells were killed because the unstable antidote is degraded faster than the toxin and leads to the postsegregational killing effect (reviewed in [19]. One example amongst others is the *ccd *addiction system (couples cell division; [20,21]) of the *Escherichia coli *F plasmid, which codes for a stable toxin (CcdB) and a less stable antidote (CcdA). CcdB inhibits GyrA, a subunit of the heterotetrameric DNA gyrase consisting of GyrA and GyrB, and thereby causes gyrase-dependent killing of the cells [22]. This inactivation can be prevented and reversed in the presence of CcdA protein. The products of treating the inactive GyrA-CcdB complex with CcdA are free GyrA and a CcdB-CcdA complex [23]. Moreover, the formation of the complex might prevent CcdA from being degraded by Lon protease in an ATP-dependent manner [24]. Though Ccd is one of the best understood addiction systems, some key mechanisms of the regulation remain unclear, presumedly because the CcdA-CcdB interaction and its stoichiometry is unexpectedly complex [25]. Eventually, upon plasmid loss, CcdB outlives CcdA and kills the cell by poisoning GyrA.

## Results

### Differences in *eGFP *expression according to variations of genetic modules

**Figure 1:**
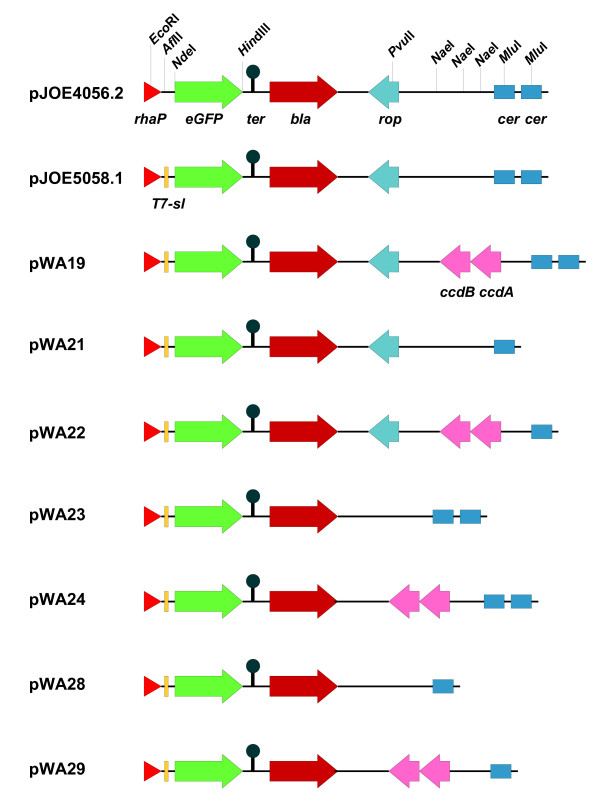
**Schematical overview of the vectors used in this study**. A physical map for relevant restriction endonucleases is given for the plasmid pJOE4056.1 and the location and orientation of the *rhaP*_*BAD *_promotor, the genes encoding *eGFP*, ampicillin resistance (*bla*), *rop *and addiction modules *ccdA *and *ccdB *are indicated by triangle and arrows. The transcription terminator sequence (*ter*) is derived from the *E. coli rrnB *operon.

In this study we tested the influence of a modification of the transcription initiation region, the presence of three genetic modules on the expression vectors and combinations thereof. The L-rhamnose-inducible vector pJOE4056.1 had already included the first 19 base pairs (bp) upstream of the AUG start codon from the highly translated bacteriophage T7 gene *10*. This sequence included a SD sequence perfectly matching the *E. coli *canonical sequence but lacked the stem-loop structure. In the first step, the original transcription initiation region of *rhaP*_*BAD *_on pJOE4056.2 was replaced by a modified T7 gene *10 *untranslated leader sequence which included the stem-loop structure. The difference to the original T7 gene *10 *upstream sequence [26] is a deletion of an 8 bp sequence between the stem-loop and SD which was replaced by the recognition site of restriction endonuclease *Afl*II. We have named the resulting promotor *rhaP*_*BAD*_-T7sl (stem-loop) and the corresponding plasmid pJOE5058.1, which was the source of the subsequent modifications. In the next step, a *ccdAB *cassette was inserted (pWA19), one of the two *cer *sites was excised (pWA21) and the *rop *site was deleted (pWA23). Plasmids with all possible combinations of these modifications were constructed (Tab. 1 and Fig. 1).

**Table 1:** Plasmids used in this study

Plasmid	Genotype		
pJOE4056.2	*rhaP*_*BAD *_*eGFP*	*cer cer rop*	Ap^R^
pJOE5058.1	*rhaP*_*BAD*_-T7sl*eGFP*	*cer cer rop*	Ap^R^
pWA19	*rhaP*_*BAD*_-T7sl*eGFP*	*cer cer rop ccdAB*	Ap^R^
pWA21	*rhaP*_*BAD*_-T7sl*eGFP*	*cer rop*	Ap^R^
pWA22	*rhaP*_*BAD*_-T7sl*eGFP*	*cer rop ccdAB*	Ap^R^
pWA23	*rhaP*_*BAD*_-T7sl*eGFP*	*cer cer*	Ap^R^
pWA24	*rhaP*_*BAD*_-T7sl*eGFP*	*cer cer ccdAB*	Ap^R^
pWA28	*rhaP*_*BAD*_-T7sl*eGFP*	*cer*	Ap^R^
pWA29	*rhaP*_*BAD*_-T7sl*eGFP*	*cer ccdAB*	Ap^R^
pWA124.1	*rhaP*_*BAD*_	*cer cer rop*	Ap^R^
pWA125.1	*rhaP*_*BAD*_-T7sl	*cer rop*	Ap^R^
pWA64.1	*rhaP*_*BAD*_-T7sl-CRP*eGFP	*cer rop*	Ap^R^
pWA73.1	*rhaP*_*BAD*_-T7sl-CRP**eGFP	*cer rop*	Ap^R^

**Figure 2:**
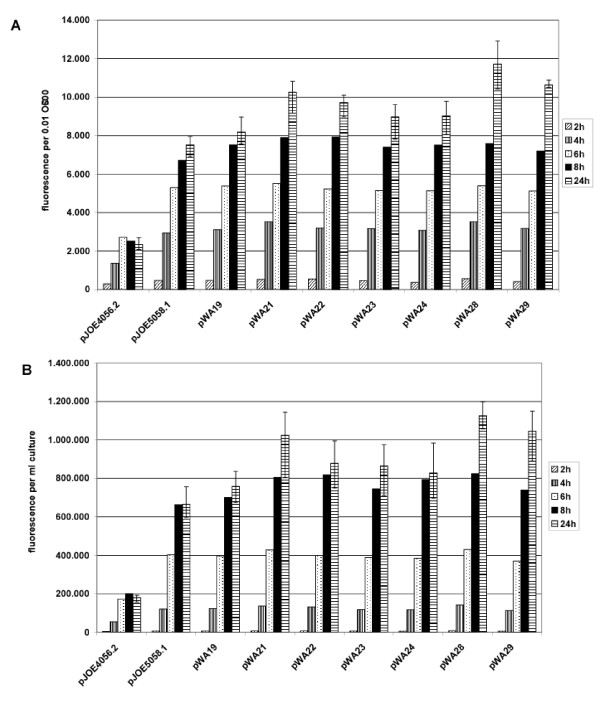
**Fluorescence intensity of eGFP in *E. coli *JM109 expressed from the indicated plasmids**. The cells were induced with 0.2% (w/v) L-rhamose and the fluorescence intensity was measured. Intensity after 2, 4, 6, 8 and 24 h are shown in (A) fluorescence per 0.01 OD_600 _and in (B) fluorescence per ml culture. Values shown are the averages of three independent experiments, for the measuring points at 24 h the standard deviation is indicated.

**Figure 3:**
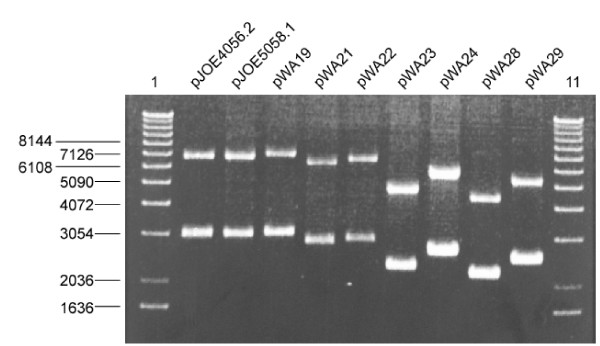
**Comparison of plasmid-DNA amounts in the *E. coli *strain JM109**. Cells were grown at 37°C in LB with ampicillin for 16 h, plasmid DNA was isolated by a boiling preparation [37] and the supernatants were analyzed by electrophoresis on a 0.5% agarose-gel and visualized by EtBr staining. Lanes 1 and 11: 1 Kb DNA Ladder (Invitrogen).

To compare the performance of the modified plasmids, the amount of eGFP produced after induction in *E. coli *JM109 transformed with the individual plasmids was measured at intervals of 60 minutes (Fig. 2). The most significant enhancement was achieved by the conversion of *rhaP*_*BAD *_to *rhaP*_*BAD*_-T7sl, whereas the insertion of *ccdAB *had only slight effects comparing pWA19 with pJOE5058.1. The fluorescence received with pJOE5058.1 is threefold that of pJOE4056.2 referring to 100 μl of a cell suspension of 0.1 OD_600 _and it is quadrupled referring to the same volume of culture. In the case of pWA22, pWA24 and pWA29 *ccdAB *even slightly decreased the expression of *eGFP*. The reduction of the two *cer *sites to one increased the measured fluorescence value, as recorded for pWA21, pWA22, pWA28 and pWA29, compared to the corresponding plasmids with a second *cer*. Eventually, the removal of *rop *again increased the production of eGFP, especially if combined with only one *cer *site, as it is the case for pWA28 and pWA29. This effect is more apparent if the values relating to fluorescence per ml culture are compared, which is due to a slightly increased optical density of these cultures (data not shown). The enhanced fluorescence achieved with the Δ*rop *plasmids correlates with an increased quantity of DNA extracted from the corresponding cultures (Fig. 3). This observation is according to expectations, as *rop *is a regulator of copy number, and a deprivation of Rop leads to an elevated copy number, in some cases concomitant with higher yields of plasmid-encoded protein. The amount of plasmid DNA extracted from strains with pWA21 and pWA22, which in contrast to pJOE5058.1 and pWA19 only contain one *cer *site, is slightly shortened. This might be due to the fact that *cer *not only is responsible for the resolution of multimers, which incidentally does not occur in *recA *strains like JM109, but also the Rcd hosted in *cer *can slow down growth of cells bearing multimers. Therefore, plasmids with two *cer *sites possibly mimic multimers, decelerate cell division and facilitate accumulation of plasmid DNA.

### Comparison of plasmid stability

**Figure 4:**
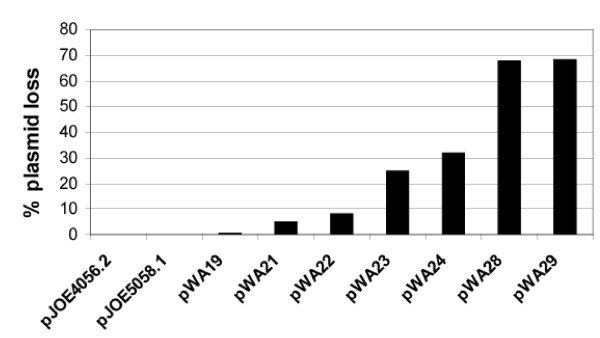
**Plasmid stability after 48 h in liquid culture without antibiotic selection**. Cells were grown at 37°C in LB supplemented with 0.2% (w/v) L-rhamnose for 24 h, starting from this culture fresh medium was inoculated, and again incubated for 24 h under the same conditions, which roughly matches 50 generations. The percentage of cells without plasmid or loss of fluorescence are shown, the values are the averages of three independent experiments.

The usability of a plasmid is not only determined by the amount of protein produced in a specific combination of strain, gene and plasmid, but also by the stability of the expression vector. Especially if the induction is carried out on a large scale and during a longer period of process, plasmid loss can have a tremendous effect on total yield. In order to test the influence of the variations of the genetic modules, we determined how many cells lost their plasmids during a prolonged induction. If the cultures were induced at 30°C for 48 or 96 hours, which is roughly equivalent to 50 generations or 100 generations, respectively, no plasmid loss could be observed in none of the cultures (data not shown). But if the induction was carried out at 37°C for 48 hours, major differences between the plasmids could be detected. The originating pJOE4056.2 and its two direct derivatives pJOE5058.1 and pWA19 were perfectly stable (Fig. 4). These results show, that the exchange of the original transcription initiation region by that of *φ10 *promoter from T7 and the insertion of the *ccdAB *locus in addition had no negative effect on plasmid maintenance. The elimination of one *cer *site producing pWA21 and pWA22 has led to a negligible raise of plasmid loss of 5–8% in approximately 50 generations, a faint disadvantage which is acceptable in regard to the enhanced expression. In contrast, the deletion of *rop *added more instability to the vectors, for instance 25% of the cells bearing pWA23 had lost their plasmids. Furthermore, the combination of Δ*rop *and *ccdAB *increased the percentage of plasmid free cells to 32% (pWA24). This observation is not according to expectations, because the addiction module should mediate programmed cell death if the gene coding for the unstable antidote is lost. Moreover, the combination of one *cer *site and Δ*rop *had a remarkably negative impact on plasmid persistence, as it resulted in a plasmid withdrawal of about 68%. Again, the addition of *ccdAB *adversely affected the plasmid stability. Apparently, combination of the specific genetic modules *cer*, *rop *and *ccdAB*, which are supposed to have a stabilizing impact on plasmids, does not simply lead to summable effects, but can contrarily destabilize vectors. In general, the influence on plasmid maintenance of plasmid-borne elements and their interaction with the particular host strain has to be examined in individual cases.

### Variations in cell length after induction of genes in dependency of *E. coli *strains

**Figure 5:**
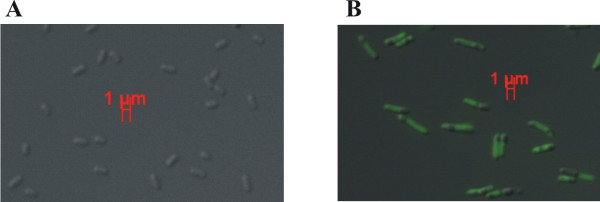
**Fluorescent light micrographs of L-rhamnose induced *E. coli *BL21 Rha^- ^without plasmid (A) and BL21 Rha^- ^with pWA21 (B)**. The bars represent 1 μm length.

**Figure 6:**
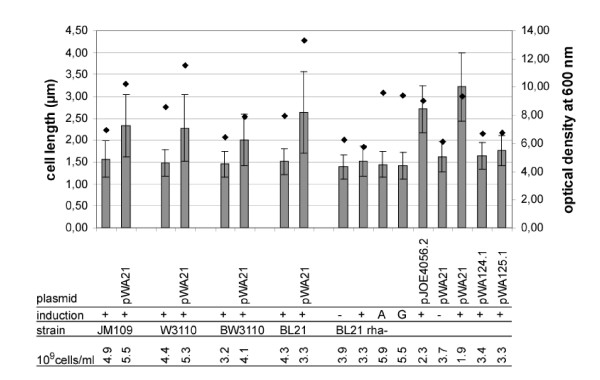
**Effect of plasmids, carbohydrates and *eGFP *expression on cell length of different strains**. (A) The strains were grown for 24 h at 30°C in LB supplemented with 0.2% (w/v) L-rhamnose (+), L-arabinose (A) or D-glucose (G) as indicated in the row 'induction'. For each culture the lengths of 100 cells were determined, the average value and the standard deviation are shown. The optical density of the cultures is indicated by a black diamond above the bar. The number of cells per milliliter of culture were determined by counting them in a Thoma-chamber.

When induction of *eGFP *was carried out in the *E. coli *strains BW3110 and BL21 Rha^- ^additionally to the experiments in JM109 mentioned above, lower values of fluorescence were observed (referring to 100 μl of a cell suspension of 0.1 OD600, data not shown). However, the relations between the fluorescence obtained with the individual vectors were comparable. But we noticed that in BW3110 and BL21 Rha^- ^the optical density of the cultures increased significantly, though these strains are not able to use L-rhamnose as a carbon source, due to the lack of *rhaB*. Examination by microscopy showed, that the reason for this raise was not a higher cell count, but an increased cell length (Fig. 5). As these differences were most significant in BL21 Rha^-^, this strain was used to examine the effect on morphology in more detail (Fig. 6).

Arabinose and glucose supplement heightened the optical density but had no effect on cell length. Moreover, no changes could be observed in cells bearing the plasmids without *eGFP *(pWA124.1 and pWA125.1) under conditions of induction, or in cells carrying the plasmid pWA21 under non-induced conditions. These experiments showed, that the increased length is not caused by the vector itself. Furthermore, the elongation was less distinct if *eGFP *was expressed from pJOE4056.2, which also achieved lower fluorescence compared to pWA21 (Fig. 2). It is quite evident, that the cell-length effect in fact is dependent on the amount of eGFP protein produced therein.

All strains showed an increase of cell length and optical density of the cultures under conditions of induction compared to cells without plasmid. The standard deviation of length in cultures with prolonged cells was larger than in the control batches, which are more homogenous. In cultures with *rhaB*-positive cells the optical density was elevated more than in *rhaB*-negative strains, an effect that is revealed in the comparison of W3110 and BW3110 or BL21 and BL21 Rha^-^. Obviously, these differences were a result of metabolization of L-rhamnose.

In the *E. coli *K12 derivatives JM109, W3110 and BW3110 the number of cells raised concomitantly to the optical densitiy if they were induced. In BL21 and BL21 Rha^-^, strains that belong to the *E. coli *B-type group, in contrast the number of cells declined if L-rhamnose was added. This effect probably is in conjunction with the stronger impact on cell length, since cell division problems might occur.

### Influence of adjusted CRP-binding site on expression of *eGFP*

As CRP is a key regulator for catabolite repression, the corresponding binding sites can be found in or near many promotors involved in carbohydrate catabolism. For instance, the *E. coli lac *promoter DNA site for CRP differs from the consensus DNA site at 7 of 22 positions. It has been shown, that CRP *in vitro *exhibits a 450-fold higher affinity for the consensus site than for the natural binding site [27].

**Figure 7:**
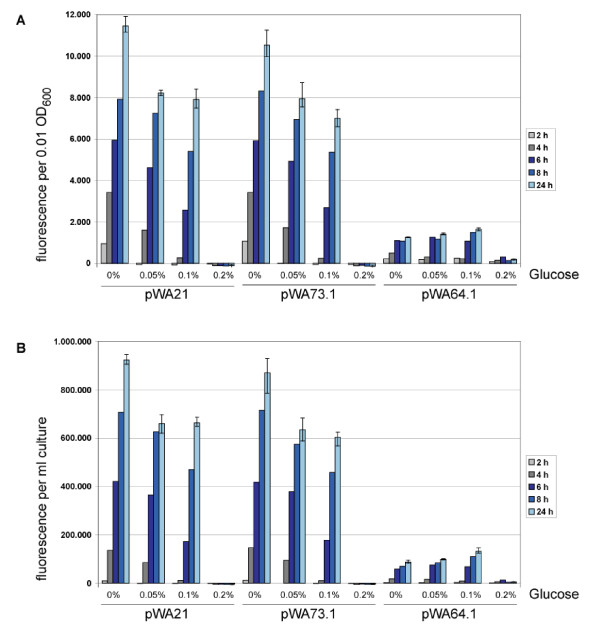
**The influence of D-glucose on the *eGFP *expression in *E. coli *JM109 with the plasmids pWA21, pWA73.1 and pWA64.1**. The cells were grown for 2 h at 37°C in LB supplemented with D-glucose as indicated, then shifted to 30°C and induced with 0.2% (w/v) L-rhamnose and the fluorescence intensity was measured. Intensity after 2, 4, 6, 8 and 24 h are shown in (A) fluorescence per 0.01 OD_600 _and in (B) fluorescence per 1 ml culture. Values shown are the averages of three independent experiments, for the measuring points at 24 h the standard deviation is indicated.

According to these observation, derivatives of pWA21 with modified CRP-binding sites were constructed. In pWA73.1 four nucleotides out of eight differing from the consensus have been adjusted, whereas in pWA64.1 one nucleotide in the most conserved region accidentally was deleted (Tab. 2). To test the influence of the mutations, the amount of eGFP produced by *E. coli *JM09 transformed with the individual plasmids, grown in liquid medium supplemented with different concentrations of D-glucose and induced with L-rhamnose, was measured (Fig. 7). The amounts of eGFP produced with pWA73.1 were comparable to those produced with pWA21. Even under conditions of D-glucose addition no differences in production were detectable, neither in the total values reached at the end of the process nor in the production rates. Obviously, the adjustment implemented in pWA73.1 did not weaken the sensitivity to catabolite repression mediated by CRP. However, the missing nucleotide in the CRP-binding site of pWA64.1 had a tremendous effect. The fluorescence was shortened to about 10% of the reference magnitude achieved by pWA21, but still it was repressed by D-glucose at a concentration of 0.2%. These results suggested that this poorly inducible promotor still is dependent on the binding of CRP, but may have a lower affinity.

**Table 2:** Sequences of the varied CRP-binding sites. The most conserved nucleotides are underlined.

Description	Sequence
consensus	AAA TGTGA TCT ▪ AGA TCACA TTT
pWA21	AAT TGTGA ACA ▪ TCA TCACG TTC
pWA64.1	AAT TGTG TCA ▪ TGA TCACA TTC
pWA73.1	AAT TGTGA TCA ▪ TGA TCACA TTC

## Discussion

Since many basic principles of genetics have been discovered using *E. coli *as a model organism, today its genetics is well characterized, and many details about the regulation of gene expression are described. This knowledge made it possible to use its promoters in a cassette fashion, unaffected by the surrounding nucleotide context. Additionally, a large number of cloning vectors are available, which provides a long-range repertory of components out of which the most appropriate can be chosen. The productivity of the expression system is influenced by many parameters, and besides the basic choice of an available expression system, these parameters can be tuned by the insertion or deletion of genetic modules.

In all organisms gene expression is regulated on various levels, at which mRNA stability provides a key control device. As several RNases are involved in mRNA degradation, including endonucleases and 3'exonucleases, many sequences that act as stabilizers have been identified. Some of them only work under specific conditions, others stabilize mRNAs of miscellanous sources under normal conditions and at high growth rates. One well characterized example of a such a stabilizing element is a 21-bp fragment of the *φ10 *promoter of bacteriophage T7 which can potentially form an 8-bp stem loop [28,29]. In our assays, the insertion of this stem loop quadrupled the amount of GFP that is produced in 24 h and fortunately had no measurable effect on plasmid maintenance. This advantage is preserved even if different combinations of other modules are added, and it exhibits the most explicit improvement achieved by the modulation that were carried out in this study. Though we have not tested the actual stability of the mRNA produced by our system, one can presume that this characteristic has been improved considerably and marks a significant step.

Besides the stem-loop insertion into the *rhaP *region we have tried to improve expression by modification of the -10 promotor region. The sequence 'TAGACT' in pJOE4056.2 has been mutated into 'TATAAT', and the resulting plasmid pJOE5115.1 has been examined in an *eGFP *expression assay as described herein. Unfortunately, this modulation resulted in a elevated basal level which under non-induced conditions attained the output of induced pJOE4056.2 cells, though it evidently fell behind in expression compared to pJOE5058.1 (data not shown). Hence, this approach reveals that additional alterations of the already efficient *rhaP*_*BAD *_expression vectors are crucial, not only because they possibly do not advance yields, but also may disturb a well-balanced system by making it less regulable.

Adjacent to plasmid inherent regulation mechanisms, the current metabolic status of the cells has an exceeding impact on the production of recombinant proteins as well. For example, some promotors act mainly in the exponential phase of cell growth, whereas others are activated primarily in steady state cultures, and in addition the intracellular availability of nutrients further affects the activity of a given promotor. Generally, promotors assigned to operons associated with carbohydrate catabolism are controlled by CRP via the intracellular cAMP levels. As D-glucose lowers cAMP levels and thus inactivates binding of CRP, but usually is added as a carbon source in minimal medium used for fermentations, it would be of interest to make such a promotor less sensitive towards low cAMP levels. The approach tested in this study was to enhance the affinity of the CRP-binding site. Unfortunately, the adjustment of the DNA sequence according to the consensus sequence had no measurable effects. The mutated plasmid still was inhibited by glucose in the same extend as the unmodified plasmid. Presumedly, the original binding site already shows a good affinity to CRP and could not be improved by an adjustment with the consensus sequence. On the other hand, the plasmid pWA64.1 with the one base pair deletion in the CRP binding site was clearly inferior. It still was susceptible to glucose addition but obtained only about 10% of the yield compared to the original plasmid. These results precisely show, that it is difficult to elevate protein production concomitantly with a low basal level and a tight regulation.

Another way to elevate protein production is to increase the corresponding gene copies in the cells. This was acchieved in this study by deleting the *rop *gene which is involved in controlling plasmid copy numbers. The deletion increased the content of plasmid DNA in the cells at least threefold as expected but there was no concomitant increase of eGFP production. This is not surprising since the eGFP production in cells with pWA21 already amounts to about 20% of the total protein and even with the strongest expression systems there is a limit at about 30% of the total protein whatever gene is used. To see the high-copy plasmid effect a reporter gene would be needed which is expressed at a lower level. This might also answer the question if there is a sufficient supply of the activator RhaS for high-copy plasmids from the single *rhaS *gene copy on the chromosome. When the *rhaRS *genes were introduced on a compatible plasmid with a moderate copy number (pBBR1MCSII, [30]) in cells containing already pWA21, pWA23 or pWA28, no significant increase in eGFP production was observed upon induction with rhamnose (data not shown).

Previous results reported by Wilms *et al.*[31] showed that the introduction of the *cer *site of ColE1 to plasmids reduces the appearance of multimers drastically. This is especially true for *recA*-proficient strains like W3110, whereas the multimerization is inhibited in *recA*-deficient strains like JM109. Actually, the insertion of *cer *led to a tremendous stabilization. Over 90% of the cells were still carrying the plasmid, while less than 50% of the cells kept the plasmids without the *cer*-site when ampicillin was absent. Accidentally, in these cases two *cer*-sites in tandem had been ligated into the vectors. The removal of one of them led to an increased eGFP expression while the plasmid stability was only negligibly influenced. The accompanying decrease in plasmid DNA apparently did not adversely affect the protein yields, probably because it is the duplication of *cer *that causes problems to the cell division which are solved by removing one. Furthermore, one *cer *site sufficiently ensured the plasmid maintenance.

Additionally, the bacterial plasmid addiction system *ccdAB *was provided to some of the vectors in order to test its influence on the stability. Unexpectedly, *ccdAB *did not deliver any improvement, as it did not stabilize the plasmid but in contrast increased plasmid loss at 37°C. As the toxin CcdB kills cells that have lost their plasmids and therefore can not produce the unstable antidote CcdA any more, apparently cells still carrying *ccdAB *plasmids are at a serious disadvantage in cell growth. It amounts to a situation where few cells that have successfully eliminated the plasmids start to overgrow cells that still express *ccdAB*. Presumedly, the continous production of the toxin becomes such a great metabolic burden to the cells, that the evasion by plasmid loss outweighs the initial toxication. Thus it appears that the insertion of the addiction module in this case represents a set-back.

## Conclusion

The initial plasmid pJOE4056.2, which already bears a well-working translation initiation signal of T7 gene *10 *and the transcription terminator from the *rrnB *operon, has been mainly improved by insertion of a stem loop and by deletion of one *cer *site. The resultant plasmid pWA21 therefore seems to perform best for *eGFP *expression in our system. Further changes might be critical as they carry the risk of worsen it. Hence, it seems as there hardly will be any room for further improvements.

## Methods

### Bacterial strains and growth conditions

*E. coli *JM109 [32] was used as a host for the cloning experiments, the measurement of fluorescence intensity, plasmid stability experiments and microscopy. Additionally, *E. coli *W3110 [33], the *rhaB*^- ^derivative BW3110, BL21 [34] and the *rhaB*^- ^derivative BL21 Rha^- ^were used for microscopy. LB liquid and LB agar plates were used as complete medium [35], supplemented with 100 μg ml^-1 ^ampicillin. For induction of the *rhaP*_*BAD *_promoter, sterile filtered L-rhamnose was added as indicated.

### General methods

Restriction enzymes and DNA modifying enzymes were purchased from Roche Applied Science (Mannheim, Germany). For restriction enzyme analysis and cloning experiments, standard methods were used [36]. Plasmid DNA was isolated according to a published protocol [37]. *E. coli *was transformed with plasmid DNA as described by Chung *et al.*[38]. All changes made in the expression vectors during this study were confirmed by DNA sequencing of the corresponding regions.

### Construction of plasmids

Table 1 lists the plasmids used in this study, Figure 1 gives a schematical overview.

Plasmid pJOE4056.2 is a fully sequenced expression vector derived from pJOE3075 [39] containing *eGFP *as a reporter gene, which is positively controlled by the inducible *rhaBAD *promoter (*rhaP*_*BAD*_) together with the CRP-cAMP binding site, a ribosomal binding site and a transcription terminator from the *rrnB *operon. The plasmid pJOE5058.1 was made by restriction endonuclease digestion of pJOE4056.2 with *Eco*RI and *Afl*II, and the 5297-bp fragment was then ligated to the oligonucleotides S3977 (5'-AAT TCA GGC GCT TTT TAG ACT GGT CGT AGG GAG ACC ACA ACG GTT TCC CTC TAG AAA TAA TTT TC-3') and S3978 (5'-TTA AGA AAA TTA TTT CTA GAG GGA AAC CGT TGT GGT CTC CCT ACG ACC AGT CTA AAA AGC GCC TG-3'), in order to replace the original transcription initiation region by that of the gene *10 *from bacteriophage T7 (*rhaP*_*BAD*_-T7sl).

The *ccd *locus of plasmid F consisting of the two genes *ccdA *and *ccdB *was amplified by PCR using the primers S3991 (5'-GGC GCG CTG ATT TGT GCG GCA TAA-3') and S3992 (5'-GGC TGC CCG GCA GAA TAC ACT GCC-3'). A lysate of *E. coli *JM109 obtained by boiling and subsequent centrifugation was used as a template. The 667-bp fragment was treated with Klenow enzyme to remove overhanging nucleotides at the 3'-ends and inserted into the positive selection vector pJOE4780.1, which was cut with the restriction endonuclease *Nae*I, to get pWA17.2. The DNA sequence was verified by sequencing with the primers S3767 (5'-TAA TAC GAC TCA CTA TAG GG-3') and S3768 (5'-ATT TAG GTG ACA CTA TAG-3'). The plasmid pWA19 was received by cleaving pWA17 with *Nae*I and ligating the *ccdAB *containing fragment into pJOE5058.1, which was cut with the same restriction enzyme.

In order to remove one of the two *cer *regions, the plasmids pJOE5058.1 and pWA19 were cut with *Mlu*I, a 264 bp-fragment was excised, the vectors were religated to obtain the plasmids pWA21 and pWA22, respectively. For the construction of the plasmids pWA23 and pWA28 the *rop *locus was eliminated by restriction endonuclease digestion with *Pvu*II and *Nae*I and religation of pJOE5058.1 and pWA21, respectively. Similarly, the *rop *locus of pWA19 and pWA22 was excised by cutting with *Pvu*II and *Kpn*I, removing the protruding 3'-overhang with Klenow enzyme, and religating the DNA to get the plasmids pWA24 and pWA29, respectively. The plasmids pWA124.1 and pWA125.1 were made by restriction endonuclease digestion of pJOE4056.2 and pWA21, respectively, with *Eco*RI and *Afl*II, a subsequent Klenow fill-in of the recessed 3'-ends and religation.

To study the influence of the CRP-binding site, the promoter region of pWA21 was cut out with the restriction endonucleases *Mlu*I and *Xba*I and the fragment was ligated into pIC20HE, which was cut with the same restriction enzymes, to obtain the plasmid pWA58.1. A site-directed mutagenesis was performed by replicating both strands in a PCR reaction with *Pfu *DNA polymerase using the primers S4113 (5'-CAG CAA ATT GTG ATC ATG ATC ACA TTC ATC TTT CCC TGG TTG CC-3') and S4114 (5'-GGC AAC CAG GGA AAG ATG AAT GTG ATC ATG ATC ACA ATT TGC TG-3'), which do not only change the nucleotide sequence in the CRP-binding site but additionally introduce a new restriction site for *Bsp*HI. The template was degraded by a digest with *Dpn*I, an enzyme which cuts only *dam *methylated DNA and leaves the newly synthesized untouched. The nicked vector DNA bearing the desired mutation then was transformed into *E. coli *JM109 via electroporation. Colonies were picked, the DNA was isolated and tested in a *Bsp*HI digest, then the CRP-binding region of the plasmids pWA61.12 and pWA70.7 was sequenced with the oligonucleotide S4083 (5'-GGC TCG TAT GTT GTG TGG-3') which binds in *lacZ*. The modified CRP-binding regions were reintroduced into the pWA21 context by means of cloning, resulting in the plasmids pWA64.1 and pWA73.1. The sequence in pWA73.1 is adjusted to the consensus sequence, whereas pWA64.1 differs from this sequence as one nucleotide is missing.

Table 2 lists the sequences of CRP-binding sites in the described plasmids, comparing the consensus sequence with the original *rhaP*_BAD _sequence and the modified ones.

### Measurement of fluorescence intensity

20 ml LB in a sterile 100 ml flask were inoculated with 200 μl of an overnight culture and incubated at 37°C for 2 h shaking with approximately 200 rpm. Then the cultures were induced with 0.2% (w/v) L-rhamnose and shifted to 30°C. Samples were taken every 60 min, and the OD_600 _was measured. The samples were diluted to an OD_600 _= 0.1 with LB. For the measurement of fluorescence, three aliquots of 100 μl of each cell suspension were added to 96 well flat-bottom polystyrene microplates (Greiner, Germany), and the fluorescence was measured with a GENios fluorometer (Tecan, Austria), at an excitation wavelength of 485 nm and an emission wavelength of 535 nm. The background fluorescence was determined by using *E. coli *JM109 cultures without plasmid.

### Determination of plasmid stability

20 ml LB with 0.2% (w/v) L-rhamnose in a sterile 100 ml flask were inoculated with approximately 1000 cells derived from an overnight culture grown in LB_amp _and incubated at the indicated temperature for 24 h shaking with roughly 200 rpm. 1000 cells of this induced culture were used to inoculate the following one, again 20 ml LB with 0.2% (w/v) L-rhamnose, and incubated for another 24 h. The OD_600 _of the culture was measured, the samples were diluted in LB and plated on LB agar. The plates were incubated for 12 h at 37°C, then 100 colonies of each sample were stroked on LB_amp _agar with 0.2% (w/v) L-rhamnose and again incubated for 12 h at 37°C.

The estimation of 1000 cells is based upon the assumption that one ml culture of an OD_600 _= 1 matches 10^9 ^cells.

### Microscopy

An overnight culture was diluted 1:100 and the cells were grown in 20 ml LB for 24 h at 30°C with 0.2% (w/v) L-rhamnose, L-arabinose or D-glucose where indicated. A drop of the living culture was placed between a microscope slide and a cover glass. The cells were viewed immediately with a Plan-APOCHROMAT 100×/1,4 oil dic objective on a Zeiss Axioplan 2 with Immersol™ 518F (Zeiss, Germany), using a combination of UV and visible (phase-contrast) light. Photographs were captured with a monochrome cooled CCD camera (AxioCam MRm, Zeiss, Germany) controlled by AxioVision 3.1. For each culture the lengths of 100 cells were determined.

## Authors' contributions

AW and TS participated in the planning of experiments, carried out the experimental work and AW drafted the manuscript. The initial idea came from JA. He was involved in the planning and analysis of the experiments and helped to draft the manuscript.

All authors read and approved the final manuscript.

## References

[B1] Khalameyzer V, Fischer I, Bornscheuer UT, Altenbuchner J (1999). Screening, nucleotide sequence, and biochemical characterization of an esterase from Pseudomonas fluorescens with high activity towards lactones. Appl Environ Microbiol.

[B2] Wilms B, Wiese A, Syldatk C, Mattes R, Altenbuchner J, Pietzsch M (1999). Cloning, nucleotide sequence and expression of a new L-N-carbamoylase gene from Arthrobacter aurescens DSM 3747 in E. coli. J Biotechnol.

[B3] Volff JN, Eichenseer C, Viell P, Piendl W, Altenbuchner J (1996). Nucleotide sequence and role in DNA amplification of the direct repeats composing the amplifiable element AUD1 of Streptomyces lividans 66. Mol Microbiol.

[B4] Egan SM, Schleif RF (1993). A regulatory cascade in the induction of rhaBAD. J Mol Biol.

[B5] Cohen SN, McDowall KJ (1997). RNase E: still a wonderfully mysterious enzyme. Mol Microbiol.

[B6] Emory SA, Belasco JG (1990). The ompA 5' untranslated RNA segment functions in Escherichia coli as a growth-rate-regulated mRNA stabilizer whose activity is unrelated to translational efficiency. J Bacteriol.

[B7] Lopez PJ, Dreyfus M (1996). The lacZ mRNA can be stabilised by the T7 late mRNA leader in E coli. Biochimie.

[B8] Shine J, Dalgarno L (1974). The 3'-terminal sequence of Escherichia coli 16S ribosomal RNA: complementarity to nonsense triplets and ribosome binding sites. Proc Natl Acad Sci USA.

[B9] Ringquist S, Shinedling S, Barrick D, Green L, Binkley J, Stormo GD (1992). Translation initiation in Escherichia coli: sequences within the ribosome-binding site. Mol Microbiol.

[B10] Summers DK, Beton CW, Withers HL (1993). Multicopy plasmid instability: the dimer catastrophe hypothesis. Mol Microbiol.

[B11] Summers DK, Sherratt DJ (1984). Multimerization of high copy number plasmids causes instability: CoIE1 encodes a determinant essential for plasmid monomerization and stability. Cell.

[B12] Patient ME, Summers DK (1993). ColE1 multimer formation triggers inhibition of Escherichia coli cell division. Mol Microbiol.

[B13] Rowe DC, Summers DK (1999). The quiescent-cell expression system for protein synthesis in Escherichia coli. Appl Environ Microbiol.

[B14] Balding C, Blaby I, Summers D (2006). A mutational analysis of the ColE1-encoded cell cycle regulator Rcd confirms its role in plasmid stability. Plasmid.

[B15] Patient ME, Summers DK (1993). ColE1 multimer formation triggers inhibition of Escherichia coli cell division. Mol Microbiol.

[B16] Sharpe ME, Chatwin HM, Macpherson C, Withers HL, Summers DK (1999). Analysis of the CoIE1 stability determinant Rcd. Microbiology.

[B17] Twigg AJ, Sherratt D (1980). Trans-complementable copy-number mutants of plasmid ColE1. Nature.

[B18] Cesareni G, Muesing MA, Polisky B (1982). Control of ColE1 DNA replication: the rop gene product negatively affects transcription from the replication primer promoter. Proc Natl Acad Sci USA.

[B19] Engelberg-Kulka H, Glaser G (1999). Addiction modules and programmed cell death and antideath in bacterial cultures. Annu Rev Microbiol.

[B20] Ogura T, Hiraga S (1983). Mini-F plasmid genes that couple host cell division to plasmid proliferation. Proc Natl Acad Sci USA.

[B21] Jaffe A, Ogura T, Hiraga S (1985). Effects of the ccd function of the F plasmid on bacterial growth. J Bacteriol.

[B22] Bernard P, Couturier M (1992). Cell killing by the F plasmid CcdB protein involves poisoning of DNA-topoisomerase II complexes. J Mol Biol.

[B23] Bahassi EM, O'Dea MH, Allali N, Messens J, Gellert M, Couturier M (1999). Interactions of CcdB with DNA gyrase. Inactivation of GyrA, poisoning of the gyrase-DNA complex, and the antidote action of CcdA. J Biol Chem.

[B24] Van Melderen L, Thi MH, Lecchi P, Gottesman S, Couturier M, Maurizi MR (1996). ATP-dependent degradation of CcdA by Lon protease. Effects of secondary structure and heterologous subunit interactions. J Biol Chem.

[B25] Dao-Thi MH, Charlier D, Loris R, Maes D, Messens J, Wyns L (2002). Intricate interactions within the ccd plasmid addiction system. J Biol Chem.

[B26] Dunn JJ, Studier FW (1983). Complete nucleotide sequence of bacteriophage T7 DNA and the locations of T7 genetic elements. J Mol Biol.

[B27] Ebright RH, Ebright YW, Gunasekera A (1989). Consensus DNA site for the Escherichia coli catabolite gene activator protein (CAP): CAP exhibits a 450-fold higher affinity for the consensus DNA site than for the E. coli lac DNA site. Nucleic Acids Res.

[B28] Mertens N, Remaut E, Fiers W (1996). Increased stability of phage T7g10 mRNA is mediated by either a 5'- or a 3'-terminal stem-loop structure. Biol Chem.

[B29] Rosenberg AH, Lade BN, Chui DS, Lin SW, Dunn JJ, Studier FW (1987). Vectors for selective expression of cloned DNAs by T7 RNA polymerase. Gene.

[B30] Kovach ME, Phillips RW, Elzer PH, Roop RM, Peterson KM (1994). pBBR1MCS: a broad-host-range cloning vector. Biotechniques.

[B31] Wilms B, Hauck A, Reuss M, Syldatk C, Mattes R, Siemann M (2001). High-cell-density fermentation for production of L-N-carbamoylase using an expression system based on the Escherichia coli rhaBAD promoter. Biotechnol Bioeng.

[B32] Yanisch-Perron C, Vieira J, Messing J (1985). Improved M13 phage cloning vectors and host strains: nucleotide sequences of the M13mp18 and pUC19 vectors. Gene.

[B33] Hill CW, Harnish BW (1981). Inversions between ribosomal RNA genes of Escherichia coli. Proc Natl Acad Sci USA.

[B34] Studier FW, Moffatt BA (1986). Use of bacteriophage T7 RNA polymerase to direct selective high-level expression of cloned genes. J Mol Biol.

[B35] Luria SE, Adams JN, Ting RC (1960). Transduction of lactose-utilizing ability among strains of *E. coli *and *S. dysenteriae *and the properties of the transducing phage particles. Virology.

[B36] Sambrook J, Fritsch EF, Maniatis T (1989). Molecular cloning: a laboratory manual.

[B37] Berghammer H, Auer B (1993). "Easypreps": fast and easy plasmid minipreparation for analysis of recombinant clones in E. coli. Biotechniques.

[B38] Chung CT, Niemela SL, Miller RH (1989). One-step preparation of competent Escherichia coli: transformation and storage of bacterial cells in the same solution. Proc Natl Acad Sci USA.

[B39] Stumpp T, Wilms B, Altenbuchner J (2000). Ein neues L-Rhamnose-induzierbares Expressionssystem für *Escherichia coli*. BIOspektrum.

